# Clustering analysis of acute mountain sickness susceptibility among young adults during rapid ascent using low‐altitude step test data

**DOI:** 10.14814/phy2.71091

**Published:** 2026-09-04

**Authors:** Shanshan Wei, Linhong Ji, Kaiqi Liu, Yijia Lu

**Affiliations:** ^1^ State Key Laboratory of Tribology in Advanced Equipment, Department of Mechanical Engineering Tsinghua University Beijing China

**Keywords:** acute mountain sickness, clustering analysis, step test, unsupervised learning

## Abstract

Acute mountain sickness (AMS) compromises health and work efficiency after rapid ascent. This study aimed to explore a low‐altitude‐data‐based method for classifying AMS‐susceptible young adults. Forty‐four low‐altitude residents (18–31 years) completed a step test at 50 m. Measurements included maximal oxygen uptake (V̇O_2_max), peripheral oxygen saturation at rest (re_SpO_2_), exercise (ex_SpO_2_), and recovery (rec_SpO_2_), heart rate at rest (re_HR), exercise (ex_HR), and recovery (rec_HR), and step index. Participants ascended to 3650 m within 6 h, and AMS was assessed using the 2018 Lake Louise Score (LLS). Associations with LLS were examined before k‐means clustering. Neither re_SpO_2_ nor re_HR was significantly correlated with LLS, whereas non‐resting‐state variables (V̇O_2_max, ex_SpO_2_, rec_SpO_2_, ex_HR, rec_HR, and step index) were. Clustering using V̇O_2_max, ex_SpO_2_, rec_SpO_2_, and step index showed strong structure (silhouette coefficient = 0.767) and 93.18% agreement with LLS. Step index and ex_SpO_2_ showed the greatest between‐cluster separation, followed by rec_SpO_2_ and V̇O_2_max. Exercise‐ and recovery‐phase variables from a low‐altitude step test may classify AMS susceptibility in young adults via unsupervised learning. Evaluated after rapid ascent to 3650 m, this noninvasive, low‐cost, and practical approach shows potential for pre‐ascent AMS susceptibility screening in healthy young adults.

## 1 INTRODUCTION

In recent years, a growing number of low‐altitude residents have traveled to or worked at high altitudes due to economic development. With changes in transportation, rapid ascent to elevations above 2500 m within hours has become common, and this accelerated ascent pattern significantly increases the risk of acute mountain sickness (AMS) (Basnyat & Murdoch, 2003; Luks et al., 2017). Clinical manifestations of AMS include headache, dizziness, nausea, vomiting, and fatigue; in severe cases, it may progress to high‐altitude cerebral edema (HACE) and high‐altitude pulmonary edema (HAPE) (Bärtsch & Roach, 2001; Gong et al., 2025; Hackett & Roach, 2001; Roach et al., 2018). Pre‐ascent identification of AMS‐susceptible individuals can alert residents to take preventive measures in advance, offering practical value in reducing the adverse health impacts of AMS.

High‐altitude hypoxia can be regarded as a physiological load on humans. Based on whether an external stimulus is applied, current low‐altitude methods for assessing AMS susceptibility can be classified as unloaded or loaded approaches. Unloaded approaches assess resting physiological characteristics under unstimulated conditions using noninvasive or invasive methods. Noninvasive methods primarily evaluate resting pulmonary function and heart rate variability, but findings across studies remain inconsistent and screening performance is limited (Boos, Bass, et al., 2018; Boos, Bye, et al., 2018; Guo et al., 2023; Karinen et al., 2012; Li, Liu, & Guo, 2020; Li, Xie, et al., 2020; Senn et al., 2006; Small et al., 2021). Invasive methods provide potential biological information through blood‐based and omics biomarkers, such as blood uric acid concentration (Liu et al., 2023), but require professional sampling and laboratory testing, limiting their use in rapid field screening (Yang et al., 2025). Loaded approaches, which are mainly noninvasive, assess physiological responses to hypoxic gas inhalation, simulated hypoxia, the cold pressor test, or exercise. Although some ventilatory, oxygenation, and cardiovascular responses are associated with AMS, screening performance remains inconsistent, and some approaches require specialized equipment such as hypoxic gas generators, hypobaric chambers, or exercise echocardiography systems (Burtscher et al., 2004; Long et al., 2007; Long et al., 2008; Moore et al., 1986; Wang et al., 2025), resulting in high costs and operational demands. Therefore, a noninvasive, low‐cost, and easily standardized method for low‐altitude assessment remains needed.

Compared with resting measurements, exercise loading increases metabolic oxygen demand and more clearly reveals individual differences in exercise oxygenation, recovery capacity, and cardiorespiratory reserve. The step test is a low‐cost, simple, and easily standardized submaximal exercise test with minimal personnel and space requirements, making it suitable for low‐altitude field assessment. Recent studies further support the validity and practical value of submaximal step tests for estimating aerobic capacity (Matsuo et al., 2020; Rowley et al., 2022).

Classifying individuals according to AMS susceptibility at low altitude also requires effective extraction of relevant data features. With advances in machine learning, research has progressed from single‐indicator comparisons to multidimensional modeling (Li et al., 2025; Small et al., 2021; You et al., 2016). You et al. (2016) integrated multiple physiological indicators using a learning vector quantization neural network and achieved an accuracy of 72.2%. Small et al. (2021) developed several supervised learning models based on low‐altitude pulmonary function, with accuracies of 65%–73%. Li et al. (2025) integrated clinical, proteomic, and metabolomic data using support vector machine‐recursive feature elimination (SVM‐RFE), achieving an accuracy of 88%. These supervised approaches require Lake Louise scores obtained after actual high‐altitude exposure as outcome labels for model training. When labels are unavailable or uncertain, unsupervised learning provides a useful alternative by identifying latent structures without predefined class labels (Sinaga & Yang, 2020). K‐means is simple and interpretable and has been applied to medical phenotyping and population stratification (De Craen et al., 2006; Loftus et al., 2022; Yan et al., 2018). Although it has also been used to stratify athletic populations and analyze LLS data (Liu et al., 2024; Richalet et al., 2021), few studies have combined dynamic physiological responses from a normoxic low‐altitude step test with k‐means clustering to explore AMS susceptibility stratification.

This study preliminarily explored a method for classifying healthy young adults undergoing rapid ascent according to AMS susceptibility using low‐altitude step‐test data. Its incremental contribution lies in combining a normoxic low‐altitude step test with k‐means clustering to integrate low‐cost physiological stress testing with multidimensional physiological stratification. During the standardized 12‐min protocol, heart rate (HR), peripheral oxygen saturation (SpO_2_), step index, and estimated maximal oxygen uptake (V̇O_2_max) were collected noninvasively during rest, exercise, and recovery. K‐means clustering was then used to stratify participants into AMS‐susceptible and non‐susceptible groups, and the classification was evaluated against measurements obtained after actual high‐altitude exposure. This standardized approach is non‐invasive, low‐cost, and easy to implement at low altitude. We hypothesized that clustering physiological responses to a low‐altitude step test could produce a meaningful classification structure.

## 2 METHODS

### 2.1 Participants

This study recruited 48 healthy low‐altitude dwelling adults (18–31 years) with no high‐altitude exposure history in the year before the test. Participants were recruited through convenience sampling. Recruitment advertisements were posted online, and volunteers were screened according to predefined inclusion and exclusion criteria. The exclusion criteria included knee injury affecting exercise within 6 months, cardiovascular disease confirmed by medical history and electrocardiography, chronic respiratory disease, hypertension, and use of any medications affecting cardiopulmonary function, autonomic nervous function, or oxygen metabolism within the past 3 months. During the study, one participant was excluded for taking unauthorized medications and three participants were excluded for traveling to higher‐altitude areas without permission. Ultimately, a total of 44 participants were included for statistical analysis. Daily regular physical activity levels at low altitude were assessed using the International Physical Activity Questionnaire (IPAQ).

### 2.2 Experimental protocol

The workflow of experimental protocol is shown in Figure S1. Forty‐four participants completed the step test at low altitude (50 m). SpO_2_(%) and HR (bpm) were measured using a finger clip pulse oximeter (Masimo, Radical‐7). Participants were instructed to remove nail polish (if present) and keep their hands stable without shaking during exercise. Throughout the test, the investigator observed the device readings and pulse waveforms; measurements exhibiting irregular waveforms or abrupt fluctuations were considered invalid and remeasured. The data quality control criteria were as follows: (1) heart rate outside the range of 60–200 beats per minute (Li, 2024); (2) sustained SpO_2_ below 90% without corresponding heart rate elevation (Hanning & Alexanderwilliams, 1995); (3) obvious baseline drift, artifacts, or missing signals in pulse waveforms. Measurements meeting any of the above criteria were defined as invalid. A total of five repeated measurements were performed under identical experimental conditions due to poor data quality, and only valid data were included in the final analysis.

The standardized step test (Li, Xie, et al., 2020; Li, Liu, & Guo, 2020) comprises three phases, during which physiological indicators are recorded. (1) Rest phase: Participants sit quietly for 3 min to stabilize their heart rate (HR fluctuation ±5 bpm) before the test begins. (2) Exercise phase: Participants perform a 5‐min step test at 22.5 steps per minute (Ketels et al., 2019; Ketels et al., 2020; Li, Liu, & Guo, 2020; Li, Xie, et al., 2020). Shushan's study (Shushan et al., 2023) demonstrated that a 5‐min exercise duration is sufficient to achieve a steady‐state HR. (3) Recovery phase: After the exercise ends, participants sit quietly for 4 min; HR in the last 30 s of the 2nd, 3rd, and 4th minute is recorded as P1, P2, and P3, respectively. The step height is 35 cm for females and 40 cm for males (Li, Liu, & Guo, 2020; Li, Xie, et al., 2020).

The step index (Li, Liu, & Guo, 2020; Li, Xie, et al., 2020) is calculated according to Formula (1):

(1) $$\text{Step index}=\text{time}\times 100/\left[\left(\text{P1}+\text{P2}+\text{P3}\right)\times 2\right]$$

V̇O_2max_ (mL/(kg·min)) was estimated using Formula (2) (Li et al., 1991; Li et al., 2023; Zhang et al., 2025), where ${\mathrm{HR}}_{\text{exercise}}$ refers to the immediate HR during exercise, and BW represents body weight. V̇O_2max_ was estimated via the submaximal step test. Compared with directly measurements, the estimates showed a correlation coefficient of 0.82, a slope of 0.91, and a mean error of 4.6% (Li et al., 1991). Characteristics of the formula development sample are presented in Table S3.

(2) $$\dot{V}O_{2\max}=10^{\left(0.438621-0.002626{\mathrm{HR}}_{\text{exercise}}+0.006238\mathrm{BW}\right)}\times \frac{1000}{\mathrm{BW}}$$

A total of 44 participants were flown from Beijing (50 m) to Lhasa (3650 m) on a flight (4 h 35 min), followed by a 1.5 h bus transfer to the experimental base. All arrivals were synchronized between 14:00 and 15:00. To minimize the potential impact of travel fatigue on the risk of AMS, all participants were instructed to rest adequately and avoid strenuous exercise on the day of their arrival at high altitude. AMS assessments were uniformly conducted the following morning. Assessment of AMS via the Lake Louise Score (LLS) (Roach et al., 2018) began 18 h after arrival (08:00 the next morning). AMS was defined by a LLS of ≥3, mandating the presence of headache along with at least one more of the following symptoms: gastrointestinal symptoms, fatigue/weakness, or dizziness/light‐headedness (Roach et al., 2011). Scoring was conducted in an independent space, and the researchers only had access to anonymized symptom questionnaires. Based on the enrollment questionnaire and medical‐history interview, individuals who had entered an area above 2500 m within the preceding year or had a diagnosed anxiety or depressive disorder were excluded. All participants were instructed to maintain adequate fluid intake (2–3 L of water daily is advised) and strictly refrain from alcohol consumption, caffeine intake, smoking, staying up late, and self‐administered medication throughout the study period. Women were asked about menstruation before the low‐altitude step test; those who were menstruating were rescheduled until menstruation had ended.

A safety monitoring protocol was implemented: For participants with resting SpO_2_ < 85%, sustained resting HR > 120 beats/min, or significant arrhythmia, all physical activities were immediately suspended, low‐flow oxygen therapy (1–2 L/min) was administered, and pharmacological intervention (e.g., oral acetazolamide) was initiated as needed.

### 2.3 Data analysis

#### 2.3.1 Correlation analysis between variables and AMS

Eight variables were extracted from the low‐altitude step test data of all 44 participants, including: V̇O_2max_, re_SpO_2_, ex_SpO_2_, rec_SpO_2_, re_HR, ex_HR, rec_HR, and the step index. The correlation between the above variables and AMS was evaluated to determine the variables to be incorporated into the clustering model. Spearman's test and simple linear regression were performed to evaluate the correlation between the above variables and AMS, with the LLS as the dependent variable and the other 8 variables as the independent variables.

#### 2.3.2 Dimensionality reduction and cluster analysis

To ensure comparability among parameters with different dimensions and units, the variables were normalized using *Z*‐score standardization. Principal component analysis (PCA) was then conducted on these variables, with the first and second principal components (PC1 and PC2) extracted. PCA dimensionality reduction performance was evaluated using the cumulative explained variance ratio. A cumulative explained variance ratio exceeding 80% indicated that the principal components effectively captured the core characteristics of the original data (Waggoner, 2021).

K‐means clustering analysis was performed on different groups of variables based on PC1 and PC2. Cluster centers were initialized using the K‐means++ algorithm (Arthur et al., 2007). The algorithm was run independently 50 times with random initializations, and the solution with the lowest within‐cluster sum of squares (WCSS) was selected as the final result. A fixed global random seed (rng = 42) was used for all analyses.

It should be noted that in this study, K‐means clustering was mainly used as a data structuring tool to explore inherent patterns within the experimental dataset, rather than a machine learning model for predicting outcomes of unseen samples.

#### 2.3.3 Evaluation of classification performance

Robustness of the clustering structure was evaluated using three indices: the silhouette coefficient (SC), Davies–Bouldin index (DBI), and Calinski–Harabasz index (CHI) (Chicco et al., 2025). The average silhouette coefficient comprehensively reflects the cohesion of each data point within its assigned cluster and its separation from neighboring clusters (Modak, 2024): SC ≤ 0.25 (insufficient); 0.26–0.5 (weak); 0.51–0.7 (reasonable); and ≥ 0.71 (strong). The number of clusters (k) was determined by the peak value of the SC. The DBI measures the ratio of within‐cluster dispersion to between‐cluster separation, where lower values denote superior clustering performance and a value below 1 is considered optimal (Roy & Mandal, 2014). The CHI evaluates clustering quality by calculating the ratio of between‐cluster to within‐cluster dispersion; higher values correspond to more distinct and internally consistent clusters (Tatikonda et al., 2025).

The LLS of participants was used to validate the clustering results. Classification accuracy, precision, and sensitivity were calculated by comparing the actual classifications derived from the LLS with the clustering results; the formulas are shown in Equations ((3), (4), (5)).

(3) $$\text{Accuracy}\;\left(\%\right)=\frac{\text{true positives}+\text{true negatives}}{\mathrm{all}\;\text{cases}}\times 100$$

(4) $$\text{Precision}\;\left(\%\right)=\frac{\text{true positives}}{\text{true positives}+\text{false positives}}\times 100$$

(5) $$\text{Sensitivity}\;\left(\%\right)=\frac{\text{true positives}}{\text{true positives}+\text{false negatives}}\times 100$$

#### 2.3.4 Sex‐related analyses

Baseline characteristics, AMS status, and cluster distributions were summarized by sex. Fisher's exact tests were used to assess the associations of sex with AMS status and cluster assignment, with odds ratios (ORs) and 95% confidence intervals (CIs) reported.

To determine whether the observed between‐sex mean difference in estimated V̇O_2_max drove the cluster structure, estimated V̇O_2_max was regressed on sex and the residuals replaced the original estimates. Standardization, PCA, and k‐means clustering were then repeated. Agreement before and after residualization was assessed using the ARI and the number of participants whose cluster assignment changed. The standardized mean difference (SMD) was used to describe the between‐sex difference in estimated V̇O_2_max.

#### 2.3.5 Sensitivity analyses involving estimated V̇O_2_max

As a sensitivity analysis, an alternative clustering solution excluding estimated V̇O_2_max was constructed while retaining the primary analysis. Agreement with the primary solution was assessed using the adjusted Rand index (ARI) and the number of changed assignments, and within‐cohort clustering accuracy relative to the LLS reference classification was also reported.

In addition, 1000 Monte Carlo error perturbations were performed using the mean and standard deviation of the absolute error reported by Li et al. (1991). In the primary scenario, absolute errors were simulated from a Gamma distribution, and error direction was specified according to the mean difference between estimated and measured values. Standardization, PCA, and k‐means clustering were repeated after each perturbation.

#### 2.3.6 Evaluation of cluster stability and sensitivity to algorithm choice

The robustness of the clustering results was validated using a bootstrap stability assessment method based on the Jaccard index (Yu et al., 2019). A total of 3000 bootstrap replicates were performed, and stratified sampling was applied in each resampling to preserve the original sample proportions across clusters. For each bootstrap replicate, the clustering structure was independently derived using only the sampled reference samples, and all original samples were then mapped to this derived structure. The average Jaccard index of each sample was calculated as its stability metric, and the overall stability was defined as the median of stability values across all samples.

To assess sensitivity to algorithm choice, the data were re‐clustered using fuzzy c‐means, GMMs with different covariance structures, PAM, spectral clustering, and Ward hierarchical clustering, with identical variable standardization, PCA, and a fixed number of two clusters. Agreement with the k‐means partition was evaluated using the ARI, the number of changed assignments, and within‐cohort clustering accuracy.

### 2.4 Statistical analysis

Data with a normal distribution were presented as mean ± standard deviation, whereas non‐normally distributed data were expressed as median (Q1–Q3). The Shapiro–Wilk test was used to assess the normality of the data, thereby providing a basis for selecting appropriate parametric or nonparametric tests in subsequent analyses.

#### 2.4.1 Intergroup comparison and effect size calculation

To compare differences in each physiological indicator across different clusters, the corresponding statistical methods were selected based on the distribution characteristics of the data. For normally distributed data, Levene's test was used to check for variance homogeneity: if the homogeneity assumption was met (*p* > 0.05), an independent samples *t*‐test was used to compare cluster mean differences; if not (*p* ≤ 0.05), a Welch‐corrected *t*‐test was used, with effect size calculated using Cohen's *d* and a 95% confidence interval (CI). If the data did not satisfy the normal distribution assumption, the Mann–Whitney *U* test was adopted, with effect size calculated using Cliff's δ and a 95% CI. All analyses used a significance threshold of *p* < 0.05.

#### 2.4.2 Feature contribution evaluation

One‐way ANOVA was used to calculate *F*‐values in order to quantify the contribution of each physiological indicator to the clustering analysis results within each variable group. Larger F‐values indicate higher indicator contributions in the clustering analysis.

## 3 RESULTS

### 3.1 Participant characteristics and variable associations

The characteristics of the 44 enrolled participants included in the analysis are presented in Table 1. The physical activity of the participants was predominantly moderate‐intensity, and all included participants successfully completed the low‐altitude step test.

**TABLE 1:** Participants characteristics.

Subject characteristics	Mean ± standard deviation
Participants	44 (male = 31;female = 13)
Age, y	24.05 ± 2.93
Height, cm	172.89 ± 8.58
Body mass, kg	65.19 ± 12.46
BMI, kg/m^2^	21.85 ± 2.63
Vigorous‐intensity activity, minutes/week	150.68 ± 93.14
Moderate‐intensity activity, minutes/week	256.7 ± 103.91
Light activity, minutes/week	49.09 ± 16.71
Total physical activity score, MET‐minutes/week	2394.27 ± 951.73

The LLS of the participants on the early morning of the next day after arriving at high altitude is shown in Table S1. The correlations between the physiological variables of the participants' low‐altitude step test and LLS are shown in Table 2. V̇O_2max_, ex_SpO_2_, rec_SpO_2_, and step index were significantly negatively correlated with LLS (*p* < 0.01), whereas ex_HR and rec_HR showed significant positive correlations with LLS (*p* < 0.01). In contrast, re_SpO_2_ and re_HR exhibited no significant correlation with LLS (*p* > 0.05).

**TABLE 2:** The correlations between the physiological variables of the subjects' low altitude step test and LLS.

	Mean	Standard deviation	95% confidence intervals	*r*	*p*
V̇O_2max_	42.22	1.84	(41.66, 42.78)	−0.55	*p* < 0.001
ex_SPO2	96.15	0.77	(95.92, 96.39)	−0.45	*p* < 0.01
rec_SPO2	96.98	0.74	(96.76, 97.21)	−0.32	p < 0.01
step index	51.52	5.08	(49.97, 53.06)	−0.51	*p* < 0.001
ex_HR	139.1	8.86	(136.4, 141.8)	0.47	*p* < 0.01
rec_HR	110.9	7.59	(108.6, 113.2)	0.46	*p* < 0.01
re_SPO2	97.03	0.77	(96.79, 97.26)	−0.17	*p* > 0.05
re_HR	76.52	10.95	(73.19, 79.85)	0.10	*p* > 0.05

These 6 variables, which are significantly correlated with AMS, characterize the oxygenation, cardiac autonomic regulation, and cardiorespiratory endurance of the low altitude participants. To compare the clustering accuracy obtained from different groups of low‐altitude step‐test physiological indicators, the variables were divided into 7 groups: Oxygenation (O) (*n* = 3: V̇O_2max_, ex_SpO_2_ and rec_SpO_2_); Cardiac autonomic regulation (C) (*n* = 2: ex_HR and rec_HR); Cardiorespiratory Endurance (E) (*n* = 1: step index); Combination 1 (O+C): oxygenation + cardiac autonomic regulation (*n* = 5: V̇O_2max_, ex_SpO_2_, rec_SpO_2_, ex_HR and rec_HR); Combination 2 (O+E): oxygenation + cardiorespiratory endurance (*n* = 4: V̇O_2max_, ex_SpO_2_, rec_SpO_2_ and the step index); Combination 3 (C+E): cardiac autonomic regulation + cardiorespiratory endurance (*n* = 3: ex_HR, rec_HR and the step index), and combination 4 (O+C+E): oxygenation + cardiac autonomic regulation + cardiorespiratory endurance (*n* = 6: V̇O_2max_, ex_SpO_2_, rec_SpO_2_, ex_HR, rec_HR and the step index).

### 3.2 Primary clustering results

**FIGURE 1:**
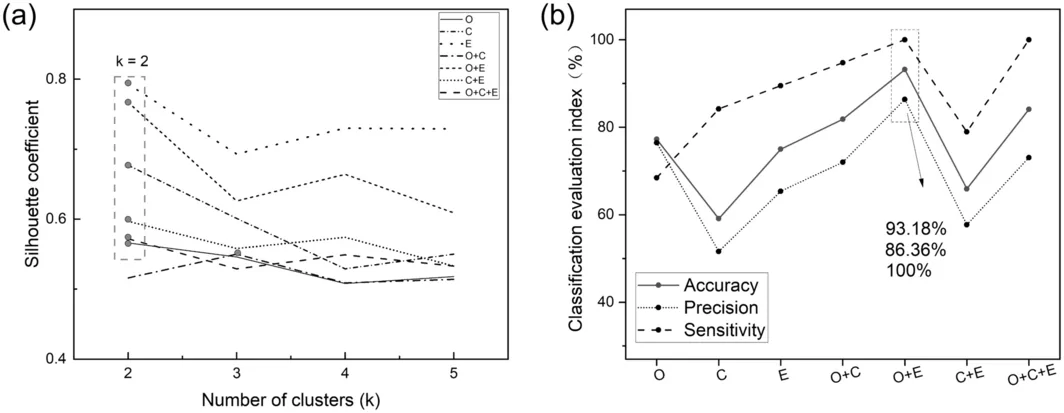
Silhouette coefficients (SC) and classification evaluation metrics of different variable groups (a) Silhouette coefficients of different variable groups across varying numbers of clusters (k). The dashed box highlights the optimal cluster number (*k* = 2) for the subsequent analysis. (b) Classification evaluation metrics (Accuracy, Precision, and Sensitivity) of different variable groups. The O+E feature set yielded the overall performance (Accuracy: 93.18%, Precision: 86.36%, and Sensitivity: 100%); these values do not represent independently validated screening performance.

Figure 1 (a) shows the SC of k‐means clustering analysis for different groups of input variables. For most groups of variables, the maximum SC was achieved at cluster number *k* = 2. Therefore, the appropriate number of classification clusters in this study was determined to be *k* = 2. The classification evaluation metrics of different variable groups are shown in Figure 1b. The clustering analysis based on combination 2 (oxygenation + cardiorespiratory endurance, *n* = 4: V̇O_2max_, ex_SpO_2_, rec_SpO_2_, step index) achieved the highest accuracy (93.18%).

The SC of combination 2 was 0.767 (strong structure), indicating high intra‐cluster compactness, strong inter‐cluster separation, and reasonable sample allocation. Based on SC analysis, clustering quality at *k* = 2 was further validated via CHI and DBI, with values of 40.598 and 0.834 respectively. Bootstrap resampling‐based clustering stability verification revealed that the median Jaccard index across all samples was 0.763.

The classification clusters based on combination 2 were compared with actual AMS incidence determined by the LLS. The corresponding labels for each cluster are presented in Table 3. 86.36% of participants in Cluster 1 had an LLS score ≥3, while all participants in Cluster 2 had LLS scores ranging from 0 to 2. All 19 participants with AMS were assigned to the AMS‐related cluster, corresponding to a within‐cohort apparent sensitivity of 100% (19/19). Table 4 presents the results of the k‐means clustering analysis based on combination 2.

**TABLE 3:** Labels corresponding to classification clusters based on combination 2.

LLS	0–2	3 and above
Cluster1	13.64%	**86.36%**
Cluster2	**100%**	0%

*Note*: Bold values indicate the predominant LLS category within each cluster and were used to assign the corresponding cluster labels.

**TABLE 4:** Results of k‐means clustering analysis based on combination 2.

Clustering analysis	True AMS	True non‐AMS	Precision
Cluster 1 (judged as AMS‐susceptible population)	19	3	86.36%
Cluster 2 (judged as AMS‐non‐susceptible population)	0	22	100%
Sensitivity	100%	88%	

### 3.3 Cluster characteristics and between‐cluster differences

**FIGURE 2:**
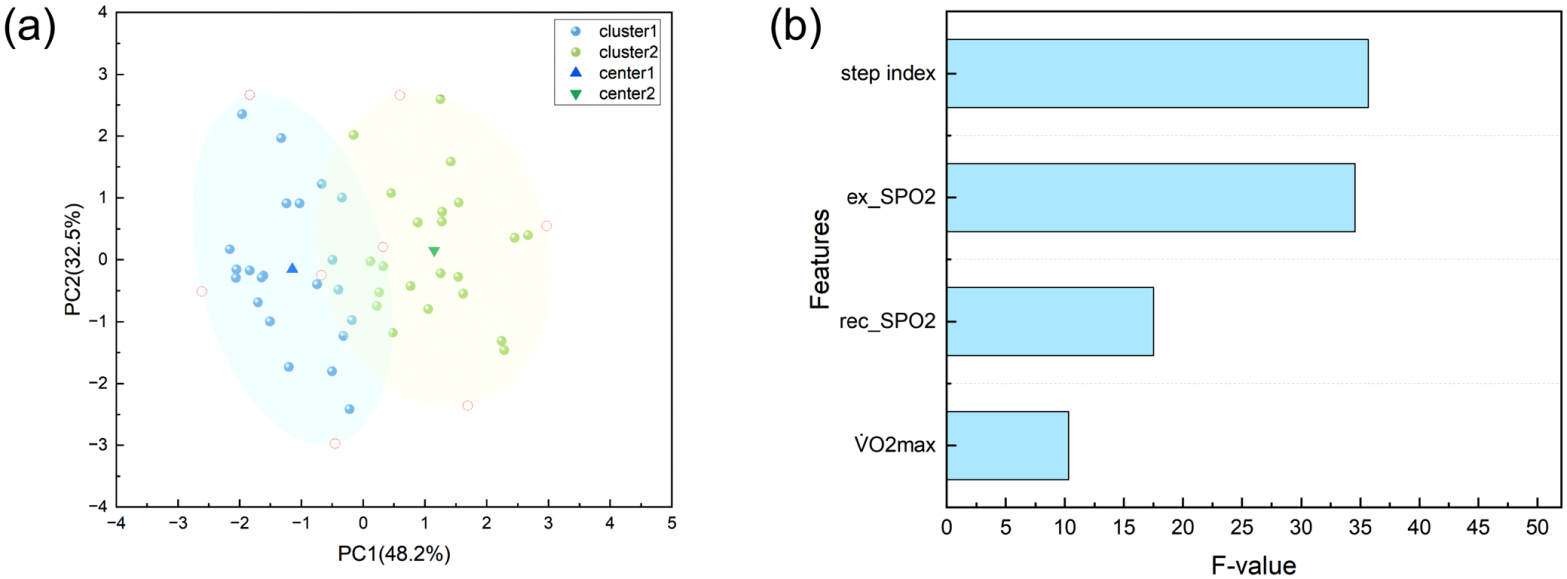
Clustering analysis results based on the oxygenation + cardiorespiratory endurance variable group (combination 2) (a) Principal component analysis (PCA) plot of the two identified clusters. The first two principal components (PC1 and PC2) explain 48.2% and 32.5% of the total variance, respectively. Ellipses indicate the estimated distribution of each cluster. (b) Contribution (*F*‐values from ANOVA) of individual variables to the cluster separation.

After dimensionality reduction using PCA, the distribution of clustering results based on combination 2 across the two principal components, PC1 and PC2, is shown in Figure 2a. The contribution of each physiological variable in combination 2 is presented in Figure 2b. The step index and ex_SpO_2_ contributed most to the separation between the two clusters, followed by rec_SpO_2_ and V̇O_2max_.

**FIGURE 3:**
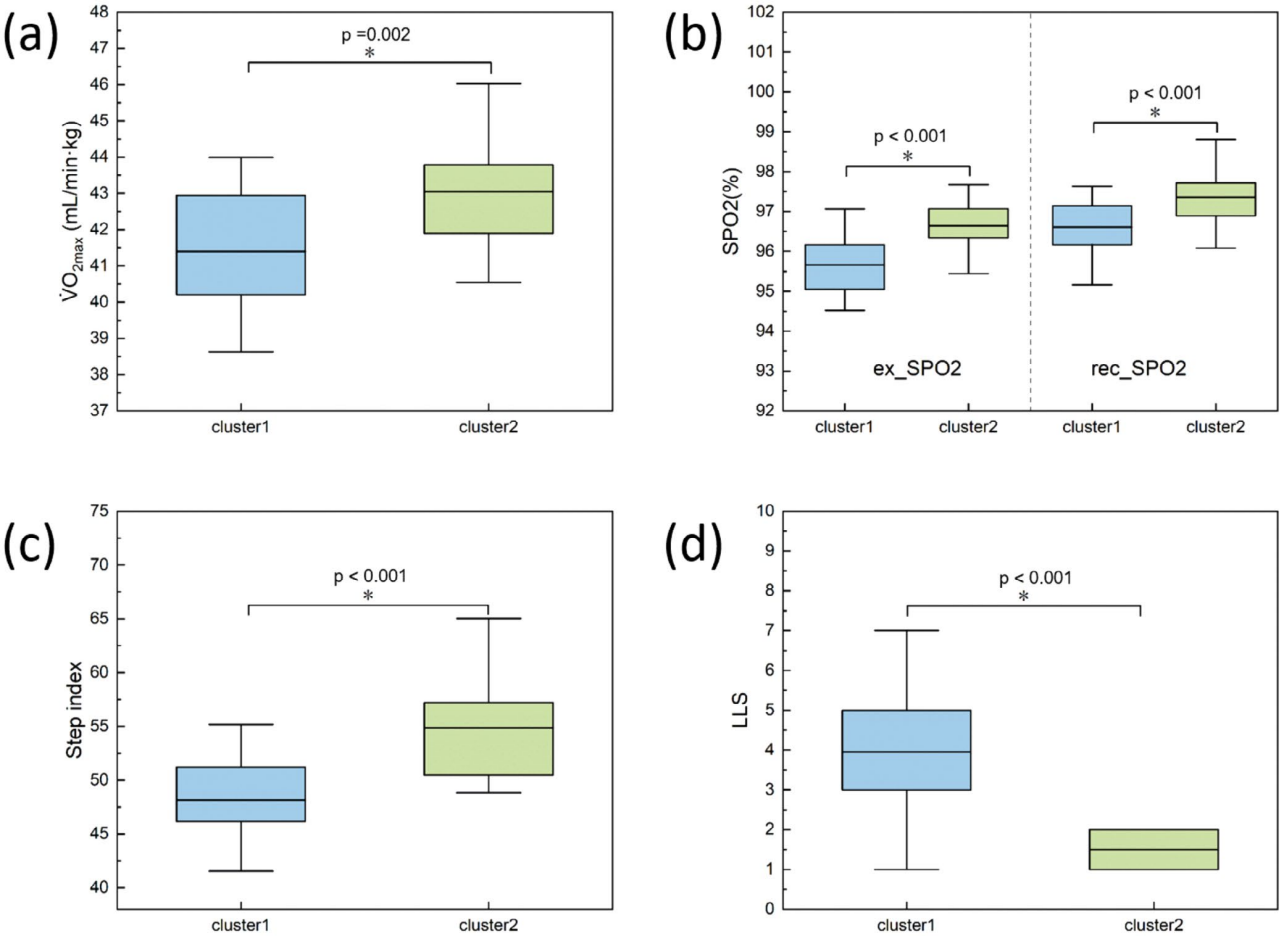
Differences in physiological variables between clusters based on combination 2 (a) low‐altitude V̇O_2_max (b) low‐altitude ex_SpO_2_ and rec_SpO_2_ (c) low‐altitude step index (d) high‐altitude LLS. *p < 0.05, independent‐samples *t*‐test. Exact *p* values from independent‐samples *t*‐tests are shown on the graph. Boxes indicate the 25th percentile, median, and 75th percentile, and whiskers indicate the range.

Participants were divided into two clusters based on the clustering results of combination 2. The four variables in combination 2 were compared to analyze the differences between different classification clusters, as shown in Figure 3a–c. Significant differences were observed between cluster 1 and cluster 2 in V̇O_2max_ (*p* < 0.01, *d* = 0.99 [0.36, 1.62]), ex_SpO_2_ (*p* < 0.001, *d* = 1.66 [0.96, 2.34]), rec_SpO_2_ (*p* < 0.001, *d* = 1.17 [0.52, 1.81]), and step index (*p* < 0.001, *d* = 1.75 [1.04, 2.44]). The LLS between the two clusters is shown in Figure 3d, with a significant difference in LLS between cluster 1 and cluster 2 (*p* < 0.001, δ = 0.82 [0.67, 0.91]). None of the 95% confidence intervals for the effect sizes contained zero, further indicating statistically significant differences between clusters.

### 3.4 Sex‐related analyses

As shown in Table S2, AMS incidence was 48.4% in men and 30.8% in women, with no statistically significant association between sex and AMS status (OR = 0.48, 95% CI: 0.09–2.20, *p* = 0.335). The proportions of men and women assigned to Cluster 1 were 51.6% and 46.2%, respectively, and sex was not significantly associated with cluster assignment (OR = 0.81, 95% CI: 0.18–3.56, *p* = 1.000). The standardized difference in estimated V̇O_2_max between men and women was small (|SMD| = 0.083). After sex residualization, all 44 participants retained their original cluster assignments (ARI = 1.000).

### 3.5 Sensitivity analyses involving estimated V̇O_2_max

Excluding estimated V̇O_2_max in the sensitivity analysis yielded moderate agreement with the primary clustering (ARI = 0.662), with 4 of 44 assignments changed. Within‐cohort clustering accuracy relative to LLS was 84.09%, versus 93.18% in the primary analysis.

Under the Gamma error scenario, the median ARI was 0.662 and the median number of changed assignments was 4. Median within‐cohort clustering accuracy and sensitivity against LLS were 84.09% and 89.47%, respectively. Results were similar under the lognormal error distribution.

### 3.6 Sensitivity to clustering algorithm choice

Fuzzy c‐means yielded the same assignments as k‐means (ARI = 1.000). PAM also showed high agreement (ARI = 0.822; 2 changed assignments). ARIs for shared‐covariance GMM, full‐covariance GMM, and spectral clustering were 0.740, 0.662, and 0.662, respectively. Complete results are provided in Table S4.

### 3.7 Methodological comparison with previous studies

As shown in Table S5, compared with previous studies, the k‐means clustering results based on the oxygenation and cardiorespiratory endurance variable group (V̇O_2max_, ex_SpO_2_, rec_ SpO_2_, and step index) proposed in this study are a noninvasive method with low time consumption (duration: 12 min), showing a clustering accuracy of 93.18% relative to the LLS reference classification (Figure 1b).

## 4 DISCUSSION

This study explored pre‐classification of AMS susceptibility in healthy young adults using a combination of low‐altitude exercise variables, thereby extending the combined assessment and analytical pathway for AMS susceptibility stratification. Conceptually, the study extended assessment from resting variables to physiological responses during exercise and recovery. Although a normoxic step test does not simulate hypoxic exposure, it increases metabolic oxygen demand and may reveal interindividual differences in exercise oxygenation, recovery, and cardiorespiratory reserve. Methodologically, estimated V̇O_2_max, exercise SpO_2_, recovery SpO_2_, and step index were integrated using k‐means clustering to explore the latent population structure. LLS labels were not used to form the clusters, and no separate diagnostic threshold was specified for any variable. Practically, the approach has limited equipment, personnel, and space requirements and may provide a potential low‐altitude method for the pre‐classification of AMS susceptibility; however, it requires validation in independent multicenter cohorts. The evaluation indicators in this study are all based on specific physiological variables related to the low‐altitude step test. The standardized low‐altitude step test has no venue restrictions, requires minimal equipment, and is simple to perform, enabling easy implementation in practical scenarios.

### 4.1 Rest phase variables VS non‐rest phase variables

As shown in Table 2, non‐rest phase physiological variables (V̇O_2max_, ex_SpO_2_, rec_SpO_2_, ex_HR, rec_HR, and step index) of the low‐altitude step test were significantly correlated with the severity of AMS, whereas rest phase variables (re_SpO_2_ and re_HR) showed weak associations. This is the key reason why the present method uses non‐rest phase data (V̇O_2max_, ex_SpO_2_, rec_ SpO_2_, ex_HR, rec_HR, and step index) rather than rest phase data (re_SpO_2_ and re_HR) for clustering. Incorporating physiological indicators measured under an exercise load is crucial for classifying individuals according to AMS susceptibility. The resting state only reflects the physiological baseline of the body under unstressed and steady‐state conditions, which hardly reveals potential defects in compensatory function. However, applying physiological stress (such as exercise) increases tissue oxygen demand, which may expose underlying issues such as insufficient ventilation and low efficiency of cardiac autonomic regulation, thereby amplifying the differences in physiological responses between AMS‐susceptible and non‐susceptible individuals. AMS stems from dysregulation or impaired compensatory function of physiological systems under hypoxic stress. Exercise‐based assessment effectively reflects overall bodily compensatory capacity under oxygen supply–demand imbalance, better simulating the physiological status of rapid high‐altitude exposure. This aligns with the results of Karinen's study (Karinen et al., 2012), where rec_SpO_2_ significantly distinguishes between AMS and non‐AMS populations, whereas re_SpO_2_ does not. The results are consistent with Liu's study (Liu et al., 2023) and Ye's study (Ye et al., 2023), whose studies found no significant difference in low altitude resting HR between high‐altitude AMS and non‐AMS groups.

### 4.2 Clustering results and classification accuracy

As shown in Figure 1a, the appropriate classification number was determined as *k* = 2 based on SC, which happens to correspond to AMS‐susceptible and non‐susceptible populations. Based on the dominant AMS prevalence status within each cluster, it can be seen in Table 3 that cluster 1 corresponds to the AMS‐susceptible population, and cluster 2 corresponds to the AMS non‐susceptible population. Combination 2 (O+E): oxygenation + cardiorespiratory endurance (*n* = 4: V̇O_2max_, ex_SpO_2_, rec_SpO_2_, step index) achieves the highest accuracy (93.18%) and SC (0.767), as shown in Figure 1b. The oxygenation variable group (O) has an accuracy of 77.27% and a SC of 0.566, while the cardiorespiratory endurance group (E) has an accuracy of 75% and SC of 0.793. The classification results of the individual variable groups are not as good as those of combination 2 (O+E). In the clustering analysis, combination 4 (O+C+E) includes cardiac autonomic regulation indicators (ex_HR and rec_HR), achieving an accuracy of 84.09% and a SC of 0.572. However, compared with combination 2 (O+E), neither the accuracy nor the SC improves, indicating that increasing the number of clustering indicators continuously does not enhance the classification results.

As shown in Figure 2a, the classification structure (two clusters) obtained from the k‐means clustering analysis of combination 2: oxygenation + cardiorespiratory endurance (*n* = 4: V̇O_2max_, ex_SpO_2_, rec_SpO_2_, and step index) is consistent with the structure of the two populations (AMS‐susceptible and AMS non‐susceptible). The confidence intervals of samples in the two formed clusters show an 18.2% overlap of samples in the PC1‐PC2 space. According to the von Luxburg criterion (the structure is valid when the overlap is <20% and SC > 0.5) (Von Luxburg, 2007), the current clustering results are valid.

As shown in Figure 2b, the step index contributes most in classifying AMS‐susceptible and non‐susceptible populations in combination 2 (O+E). Although its classification accuracy is low when used alone (75%), combining it with other variable groups improves the classification accuracy. For example, the accuracy of O+E (93.18%) is higher than that of O (77.27%); the accuracy of C+E (65.91%) is higher than that of C (59.09%); and the accuracy of O+C+E (84.09%) is higher than that of O+C (81.82%), as shown in Figure 1b.

The value of 93.18% represents only the within‐cohort clustering accuracy relative to the LLS reference classification, rather than predictive accuracy in an independent sample. The findings indicate an exploratory stratification in the non‐resting physiological data from the low‐altitude step test that is associated with AMS status.

The observed sensitivity of 100% indicates only that all 19 participants with LLS‐defined AMS in this cohort were assigned to the AMS‐related cluster. It does not imply that no false‐negative assignments would occur in a new validation cohort and should not be interpreted as validated screening sensitivity.

### 4.3 Cluster robustness and sensitivity analyses

The PC1 and PC2 accounted for 80.7% of the cumulative explained variance, indicating that they effectively captured the core characteristics of the original data. The CHI was 40.598, indicating markedly greater between‐cluster variance than within‐cluster variance and high intra‐cluster homogeneity. The DBI was 0.834, suggesting low within‐cluster dispersion, large inter‐cluster distance and minor distribution overlap between the two clusters, thus forming distinct clustering structures. Bootstrap validation based on the Jaccard index yielded a median overall clustering stability of 0.803. Values ranging from 0.75 to 0.85 indicate stable clustering performance (Cheng & Milligan, 1996), demonstrating that the identified clusters maintained high consistency under data perturbation, with most samples retaining stable cluster assignments across repeated resampling.

Across sensitivity analyses excluding estimated V̇O_2_max or introducing individual‐level estimation‐error perturbations, clustering showed moderate agreement with the primary analysis, although some assignments and concordance with LLS decreased. Thus, the primary cluster structure was not solely driven by estimated V̇O_2_max but remained somewhat sensitive to the information captured by this variable and its estimation error.

Fuzzy c‐means, PAM, and shared‐covariance GMM broadly reproduced the primary stratification identified by k‐means, providing some evidence of internal robustness. K‐means was therefore retained as the exploratory primary analysis, and the two clusters were interpreted as a physiological stratification with some internal stability.

K‐means is simple and interpretable, but its results may be influenced by the predefined number of clusters, data preprocessing, and sample composition (De Craen et al., 2006; Ikotun et al., 2023; Khan et al., 2024; Loftus et al., 2022). We therefore assessed internal robustness using data standardization, multiple initializations, bootstrap resampling, and alternative clustering algorithms. Although some algorithms reproduced similar stratification patterns, individual assignments were not entirely consistent, indicating that the two clusters should be interpreted as exploratory physiological stratification.

### 4.4 Differences between the two classification clusters

As shown in Figure 3a–d, an analysis of the differences in the four variables of combination 2 and LLS across different clusters revealed significant differences between cluster 1 and cluster 2 of combination 2 in terms of V̇O_2max_, ex_SpO_2_, rec_SpO_2_, step index, and LLS. Low‐altitude V̇O_2max_, ex_SpO_2_, rec_SpO_2_, and the step index are effective indicators for distinguishing between AMS‐susceptible and AMS non‐susceptible populations.

The three participants misclassified into cluster 1 (AMS‐susceptible) had a lower step index and ex_SpO_2_ (ranked top 2 in contribution) than cluster 2 (AMS‐non‐susceptible) participants, while their values were close to those in cluster 1. One possibility is that their low cardiorespiratory endurance and exercise oxygenation reserves led the algorithm to classify them into cluster 1 based on similarity of physiological variable. Another possibility that must be discussed is that the actual LLS results may be underestimated due to the conservative subjective scoring of the participants during the LLS assessment. Participants with conservative scoring require focused attention from medical institutions to avoid adverse health consequences caused by neglect due to their conservative scoring. The discrepancy between the low altitude clustering results and the high‐altitude LLS results should be analyzed, as it may indicate individuals with underestimated LLS; high‐altitude medical departments should pay attention to this to ensure the health of residents from lower altitudes who are ascending to high altitudes. Because the LLS is a self‐reported research instrument, symptom reporting may be influenced by individual perception and the assessment setting; underreporting of symptoms is only one possible, unverified explanation.

### 4.5 Sex and other potential confounders

Previous findings on sex and AMS susceptibility have been inconsistent. Schneider et al. surveyed 827 mountaineers after ascent to 4559 m and found that sex was not an independent risk factor for AMS. In contrast, Häfliger et al. observed a higher incidence of AMS among women in a randomized trial of healthy low‐altitude residents aged over 40 years who ascended from 760 m to 3100 m and remained there for 2 days, suggesting that sex effects may depend on the study population and exposure conditions (Häfliger et al., 2025; Schneider et al., 2002). In the present study, sex was not significantly associated with AMS status or cluster assignment, and sex residualization did not alter the clustering results. However, only 13 women were included and the confidence intervals were wide, so a sex effect cannot be excluded.

Standardized lifestyle instructions were used to reduce short‐term behavioral influences; however, sleep quality and adherence were not evaluated using validated questionnaires, sleep diaries, or objective devices. Previous research found a higher risk of AMS after rapid ascent to 3700 m among individuals with poorer subjective sleep quality (Tang et al., 2014). That study used an earlier version of the LLS that included a sleep item, whereas the 2018 revised LLS used here removed this item, reducing direct overlap between sleep and AMS assessment (Roach et al., 2018). Nevertheless, insufficient sleep may still affect heart rate, exercise performance, recovery, and perceived fatigue (Roach et al., 2018; Tang et al., 2014).

Participants with diagnosed psychiatric disorders were excluded, but transient psychological stress and anxiety were not assessed using standardized instruments. Boos et al. reported that greater anxiety proneness at low altitude may be associated with a higher risk of severe AMS and that anxiety at altitude was weakly associated with AMS symptoms (*r* = 0.31); however, the causal direction remains unclear (Boos, Bass, et al., 2018; Boos, Bye, et al., 2018). Psychological state may therefore influence exercise engagement, heart rate, perceived fatigue, and subjective LLS reporting. Although the clustering analysis primarily included objective physiological variables, psychological factors may still have affected some cluster variables and the assignment of individuals near cluster boundaries.

Women who were menstruating were rescheduled, reducing the direct influence of menstrual discomfort and fatigue on the low‐altitude exercise measurements; however, the specific menstrual‐cycle phase and hormone levels were not recorded. Recent studies have reported no clear difference in AMS incidence between the follicular and luteal phases and no significant effect of menstrual‐cycle phase on hypoxic ventilatory response, SpO_2_, or submaximal exercise responses at altitude (Citherlet et al., 2024; Gardner et al., 2024; Tagliapietra et al., 2024). Nevertheless, these studies differed from the present study in design and some had small samples, so individual‐level cycle effects cannot be excluded. Because only 13 women were included, the present study could not adequately assess the influence of menstrual‐cycle phase on the cluster structure or AMS status.

Excluding low‐altitude residents who had been exposed to altitudes above 2500 m within the preceding year reduced the potential influence of recent acclimatization. Restricting recent high‐altitude exposure is common in rapid‐ascent studies, although reported exclusion windows range from 7 days to 1 year and no uniform standard exists (Li et al., 2025; Senn et al., 2006; Wang et al., 2025). The 1‐year criterion used here, which is also used by Li et al. (2025), represents a relatively conservative approach.

### 4.6 Limitations

As this exploratory study included a small sample of young adults aged 18–31 years undergoing rapid high‐altitude ascent, of whom 29.5% were women, the cluster structure may be sensitive to sample size and sex composition. V̇O_2max_ was indirectly calculated using a formula and therefore served as an estimated value rather than being directly measured by gas analysis. All participants ascended to a high altitude of 3650 m by taking a 6‐h flight to obtain LLS data. Future studies may further explore the performance of this clustering‐based classification approach under different ascent rates. Although standardized lifestyle instructions, exclusion of participants with recent high‐altitude exposure or diagnosed psychiatric disorders, and rescheduling of women who were menstruating reduced some potential confounding, sleep quality was not objectively assessed and subclinical psychological state and specific menstrual‐cycle phase were not systematically recorded. Residual confounding of the step‐test variables and LLS assessments by unmeasured factors therefore cannot be excluded. The study examined only rapid ascent to 3650 m, and the maximum LLS was 7; severe AMS, HACE, and HAPE were not represented. Moreover, all clustering, variable‐combination comparisons, and LLS agreement assessments were conducted in the same cohort. Thus, the findings represent exploratory physiological stratification and should be extrapolated with caution to populations of different ages, altitudes, ascent profiles, or disease severity levels.

## 5 CONCLUSION

In this exploratory study, physiological stress was induced through a standardized step test at low altitude, and relevant physiological data was collected. The k‐means clustering algorithm was used to analyze the participants' physiological data, and a method of classifying AMS‐susceptible and non‐susceptible populations based on the results of low‐altitude step test was proposed. The results showed that the indicators from the exercise and recovery phases (ex_SpO_2_, rec_SpO_2_, ex_HR, rec_HR, V̇O_2max_, and step index) were significantly more correlated with LLS than the rest phase variables (re_SpO_2_ and re_HR). Therefore, the physiological indicators collected during the non‐resting phases were crucial for classifying individuals according to AMS susceptibility. The clustering analysis based on the low altitude oxygenation and cardiorespiratory endurance variable group (V̇O_2max_, ex_SpO_2_, rec_SpO_2_, and step index) generated a strong classification structure (SC = 0.767, DBI = 0.834, CHI = 40.598, Jaccard coefficient = 0.803), with significant differences in each variable and LLS between different clusters. This exploratory study showed that clustering based on exercise‐ and recovery‐phase physiological data from a low‐altitude step test yielded a within‐cohort accuracy of 93.18% relative to the LLS reference classification, providing a potential noninvasive, low‐cost, and practical approach to pre‐stratifying AMS susceptibility in healthy young adults. Future validation is required in larger and more diverse populations (e.g., individuals of different ages and health statuses).

## AUTHOR CONTRIBUTIONS

**Shanshan Wei:** Formal analysis; investigation; methodology; validation; visualization; writing – original draft; writing – review and editing. **Linhong Ji:** Conceptualization; supervision; writing – review and editing. **Kaiqi Liu:** Writing – review and editing. **Yijia Lu:** Conceptualization; methodology; resources; supervision; writing – review and editing.

## FUNDING INFORMATION

No funding information provided.

## CONFLICT OF INTEREST STATEMENT

The authors have no conflicts of interest to declare that are relevant to the content of this article.

## ETHICS STATEMENT

This study was approved by the Institutional Review Board of Tsinghua University (IRB No. 20190042) and was conducted in accordance with the Declaration of Helsinki. All participants provided written informed consent.

## Supporting information


Appendix S1.

## Data Availability

The datasets generated during and/or analyzed during the current study are available from the corresponding author on reasonable request.
