# Supplementary material for: Clustering analysis of acute mountain sickness susceptibility among young adults during rapid ascent using low‐altitude step test data
Source: Physiol Rep. 2026 Sep 4;14(17):e71091. doi: 10.14814/phy2.71091 (PMC13542986; doi:10.14814/phy2.71091)
Supplement: Supplementary file 1 [file PHY2-14-e71091-s001.docx]

Table S1 Lake Louise score (LLS) of subjects on the early morning of the next day after arriving at high altitude

|  | All | AMS group (LLS ≥ 3) | Non-AMS group (LLS < 3) |
| --- | --- | --- | --- |
| **LLS** |  |  |  |
| Median（IQR:Q1 - Q3） | 2（1-3） | 3（3-5） | 1（1-2） |
| Range (Min - Max) | 0 - 7 | 3 - 7 | 0 - 2 |
| **Distribution** | | |  |
| Sample size (n) | 44 | 19 | 25 |
| None (LLS 0-2) | 25 | / | 25 |
| Mild (LLS 3-5) | 17 | 17 | / |
| Moderate (LLS 6-9) | 2 | 2 | / |

Table S2. Sex-stratified participant characteristics, AMS status, and cluster allocation

| Characteristic | Male (n=31) | Female (n=13) | Effect estimate | P value |
| --- | --- | --- | --- | --- |
| Age, y | 24.19 ± 2.88 | 23.92 ± 2.75 | \|SMD\|=0.095 | — |
| Height, cm | 177.03 ± 6.06 | 162.69 ± 5.27 | \|SMD\|=2.454 | — |
| Body mass, kg | 70.57 ± 10.31 | 52.35 ± 6.09 | \|SMD\|=1.958 | — |
| BMI, kg/m² | 22.45 ± 2.50 | 19.74 ± 1.67 | \|SMD\|=1.182 | — |
| Estimated V̇O₂max, mL·kg⁻¹·min⁻¹ | 42.265 ± 2.012 | 42.110 ± 1.430 | \|SMD\|=0.083 | — |
| AMS status |  |  |  |  |
| AMS | 15 (48.4%) | 4 (30.8%) | OR=0.48 (95%CI: 0.09–2.20) | 0.335 |
| Non-AMS | 16 (51.6%) | 9 (69.2%) | — | — |
| Cluster allocation |  |  |  |  |
| Cluster 1 | 16 (51.6%) | 6 (46.2%) | OR=0.81 (95%CI: 0.18–3.56) | 1 |
| Cluster 2 | 15 (48.4%) | 7 (53.8%) | — | — |

Continuous variables are presented as mean ± standard deviation, and categorical variables as n (%). SMD, standardized mean difference; OR, odds ratio; CI, confidence interval. ORs represent the odds for females relative to males. P values were calculated using Fisher’s exact test.

Table S3. Characteristics of the samples used to develop and validate the original V̇O₂max estimation equation（Li 1991）

| Sample | n | Sex | Age (years) | Height (cm) | Body mass (kg) |
| --- | --- | --- | --- | --- | --- |
| Development sample | 40 | Male | 20.6 ± 1.3 | 167.7 ± 4.9 | 58.1 ± 5.1 |
| Independent validation sample | 27 | Male | 19.2 ± 1.8 | 169.7 ± 4.7 | 60.8 ± 6.6 |

Note: Data are presented as mean ± standard deviation. In the independent validation sample, the mean absolute estimation error was 1.96 ± 1.77 mL·kg⁻¹·min⁻¹, corresponding to approximately 4.6% of the mean measured V̇O₂max. V̇O₂max, maximal oxygen uptake.

Table S4. Comparison of alternative clustering algorithms with the primary k-means solution

| Clustering algorithm | Cluster sizes | ARI vs. k-means | Changed assignments, n | Within-cohort clustering accuracy (%) |
| --- | --- | --- | --- | --- |
| k-means clustering | 22/22 | 1.000 | 0 | 93.18 |
| Fuzzy c-means | 22/22 | 1.000 | 0 | 93.18 |
| PAM (k-medoids) | 20/24 | 0.822 | 2 | 88.64 |
| GMM (shared covariance) | 19/25 | 0.740 | 3 | 86.36 |
| GMM (full covariance) | 26/18 | 0.662 | 4 | 84.09 |
| Spectral clustering | 26/18 | 0.662 | 4 | 84.09 |
| Ward hierarchical clustering | 26/18 | 0.518 | 6 | 79.55 |

Note: ARI, adjusted Rand index; GMM, Gaussian mixture model; PAM, partitioning around medoids. ARI quantifies agreement between each algorithm and the primary k-means solution. Changed assignments indicate the number of participants assigned differently from k-means after accounting for arbitrary cluster-label switching. Within-cohort clustering accuracy was calculated by post hoc comparison of cluster assignments with LLS-defined AMS status; LLS was not used to form the clusters.

Table S5 Methodological characteristics of previous AMS assessment approaches

| Baseline altitude（m) | High altitude (m) | Physiological indicators | Invasive | Duration | Reference |
| --- | --- | --- | --- | --- | --- |
| 200 | 3648 | Uric Acid (UA) | Yes | 15min | Liu (2023) |
| 1,600 | 4800* | Hypoxic ventilatory response | No | 30min | Moore (1986) |
| 600 | 2000-4500* | SaO2 | No | 1h | Burtscher (2004) |
| 242 | 4500* | Blood pressure change | No | 20min | Long (2007) |
| 560 | 3675 | Blood pressure change | No | 20min | Long (2008) |
| 500 | 3600 | pulmonary vascular resistance | No | 30min | Wang (2025) |
| 150 | 2835 | Forced vital capacity | No | 20min | Anholm (1979) |
| 50 | 4000 | PNN50 and count of eosinophil | Yes | 15min | Li (2020) |
| 2400 | 3000-4300 | RMSSD | No | 13min | Karinen(2012) |
| 50 | 4000* | Hfnu and LF/HF combined factor | No | 15min | Guo (2023) |
| 800 | 3650 | HRV | No | 1h | Boos (2018) |
| 1350 | 4700 | Multiple physiological indicators | No | 1.5h | You (2016) |
| 1250 | 3810 | Pulmonary Function Test (PFT) | No | 45min | Small (2021) |
| 500 | 3650 | Plasma cytokines | Yes | 15min | Li (2025) |
| 50 | 3650 | （V̇O₂、ex_SPO2, rec_SPO2，step index） | No | 12min | This work |

* In a simulated hypobaric and hypoxic environment

**FIGURE S1** The overall workflow of experimental protocol
